# Iliac branch device for infrarenal aortic dissection: A novel off-label strategy

**DOI:** 10.1016/j.jvscit.2026.102258

**Published:** 2026-04-10

**Authors:** Diego Ardiles, Jeison Peñuela, Marcelo Lagos, Sebastián Alba, Manuel Espíndola

**Affiliations:** a Cirugía Vascular y Endovascular, Clínica Las Condes, Santiago, Chile; b Cirugía Vascular y Endovascular, Hospital San Juan de Dios, Santiago, Chile; c Cirugía Vascular y Endovascular, Redsalud Mayor, Temuco, Chile; d Servicio Cirugía Vascular, Hospital DIPRECA, Santiago, Chile

**Keywords:** Infrarenal aortic dissection, Triple lumen, Endovascular repair, Iliac branch device, Off-label use

## Abstract

Infrarenal aortic dissection is a rare condition, and the presence of a triple lumen has been described as a marker of instability and poor prognosis. We report the case of a 56-year-old woman with infrarenal dissection and triple lumen who presented with refractory abdominal pain. Owing to hostile anatomy, conventional endografts were unsuitable. An iliac branch device was used off-label as the main body, achieving complete reconstruction and preservation of bilateral iliac flow. This case expands previous off-label applications of iliac branch devices, demonstrating feasibility in infrarenal dissection beyond aneurysmal or occlusive disease.

Isolated infrarenal aortic dissection is a rare entity, with a clinical spectrum ranging from spontaneous resolution to progression with risk of rupture or ischemic complications. The presence of a triple lumen, characterized by the formation of two false lumens in addition to the true lumen, has been reported as a sign of unstable progression and poor prognosis, associated with increased risk of rupture, aneurysmal expansion, and persistent or recurrent symptoms.^1^

In hemodynamically stable patients, optimal medical management, including strict blood pressure control, is the first-line treatment. However, refractory pain, radiological progression, or high-risk features such as triple-lumen configuration warrant consideration of invasive intervention.

Particularly in patients with a complex anatomy—short or small necks, narrow bifurcations, or severely compromised true lumen—commercially available endografts may be inadequate. In this context, iliac branch devices (IBDs), originally designed to preserve hypogastric artery perfusion in common iliac aneurysms, may offer an off-label alternative for complex aortic reconstructions.^2^

We present the case of a patient with infrarenal aortic dissection with triple lumen and refractory pain, successfully treated with the off-label use of an E-liac IBD (JOTEC Gmbh, Hechingen, Germany).

## Case report

A 56-year-old woman with a history of well-controlled hypertension, presented with sudden-onset abdominal pain radiating to the lumbar region, persistent but self-limiting. Computed tomography (CT) of the abdomen revealed a focal infrarenal aortic dissection flap limited to zone 9. The patient remained hemodynamically stable, with controlled blood pressure, and was managed symptomatically.

**Fig 1:**
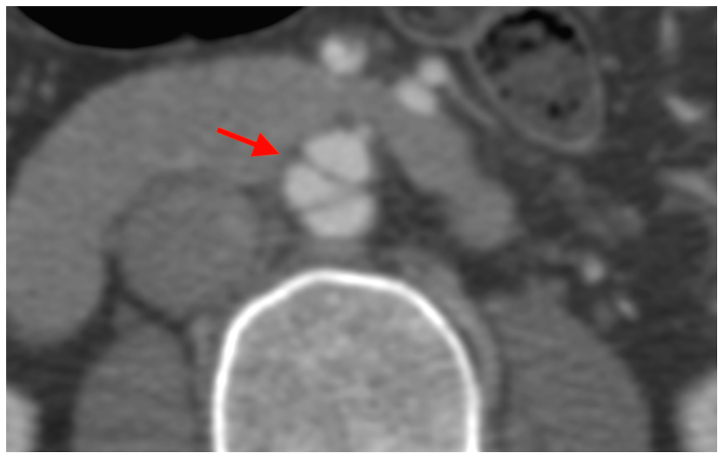
Computed tomography (CT) angiography of the current clinical presentation. The *red arrow* indicates progression of the dissection with aneurysmal dilatation and transformation into a triple-lumen configuration, also known as the Mercedes-Benz sign.

One month later, she presented again with a similar episode of sudden pain, this time with mild hypertension. Aortic CT angiography showed the previously identified dissection flap, now with progression and the appearance of a triple lumen (Fig 1). Despite renewed medical management, she developed refractory pain within 24 hours. In addition, follow-up imaging demonstrated progression of the previously known dissection, with transformation into a triple-lumen configuration. These clinical and radiological findings prompted the decision to proceed with endovascular treatment.

## Anatomical and technical considerations

Preoperative CT angiography demonstrated a severely restricted infrarenal anatomy. The proximal neck measured 13 mm in diameter and 25 mm in length, and the maximum aortic diameter at the level of the triple-lumen dissection was 15 mm, with marked true lumen compression. The distance from the lowest renal artery to the aortic bifurcation was 71 mm. These dimensions were below the minimum proximal diameter requirements of commercially available infrarenal endovascular aneurysm repair devices in our region, whose smallest main body diameter is 20 mm, with a contralateral gate length of approximately 80 mm. Given the small aortic diameter and limited bifurcation space, conventional endovascular aneurysm repair was not feasible. For this reason, an IBD was selected off-label as the main body to achieve adequate reconstruction while preserving bilateral iliac perfusion.

## Surgical technique

A right axillary approach was obtained under ultrasound guidance using a preclosure technique, and a 10F sheath was placed. The same procedure was performed in the right femoral artery. A hydrofilic guidewire and intravascular ultrasound introduced through the femoral access confirmed passage through the true lumen. This was exchanged for a support wire, followed by progressive dilation and placement of an 18F sheath.

From the axillary access, a hydrophilic guidewire and catheter were advanced into the iliac artery, and a snare was used to establish a through-and-through wire between the axillary and femoral accesses. Intravascular ultrasound examination was used again to confirm true lumen positioning. The axillary sheath was then upsized to 12F, and a pigtail catheter was positioned at the suprarenal level for angiographic guidance.

According to the preoperative plan, an IBD (E-liac 16-14-121, JOTEC Gmbh) was advanced and positioned at the infrarenal aorta, with the hypogastric gate oriented toward the contralateral iliac artery. Upper extremity access was preferred to facilitate gate cannulation given the severely restricted space at the bifurcation. Reference aortography was performed, and the device was deployed uneventfully.

Through the axillary access, an 8F sheath was advanced into the iliac branch component. The contralateral iliac artery was cannulated, and covered stents (VBX 8L-59 mm and VBX 8L-79 mm; W. L. Gore & Associates) were deployed to connect the IBD to the iliac axis. Postdilation was performed using a 10 × 40 mm balloon.

The ipsilateral branch of the IBD was then released, followed by deployment of an iliac extension (E-tegra 13 × 80 mm, JOTEC Gmbh). Balloon molding was performed on the main body and iliac branches. Final kissing balloon inflation using two 8 × 20 mm balloons ensured full expansion and symmetry at the bifurcation.

**Fig 2:**
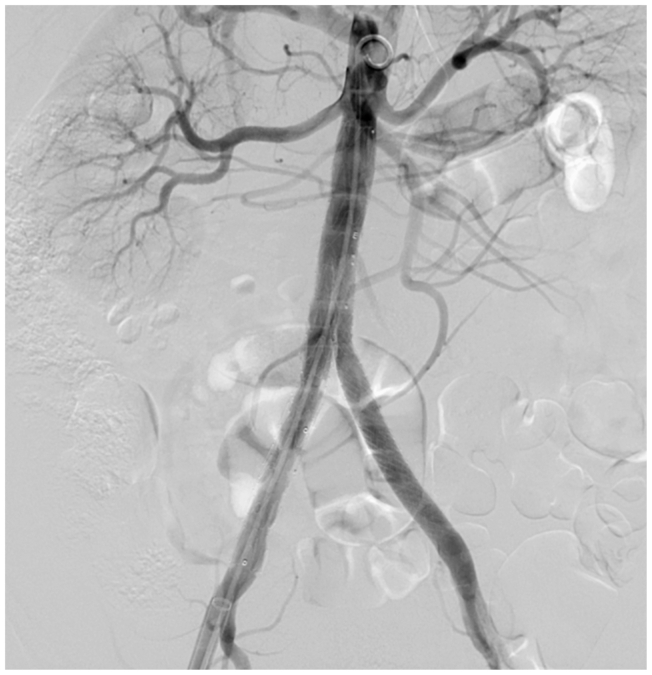
Completion aortography demonstrating successful deployment of the iliac branch device (IBD), with preserved patency of the aortic reconstruction and bilateral iliac flow, and no residual dissection.

Final angiography demonstrated a patent endograft, preserved bilateral iliac perfusion, and complete exclusion of the false lumens (Fig 2).

## Postoperative course

**Fig 3:**
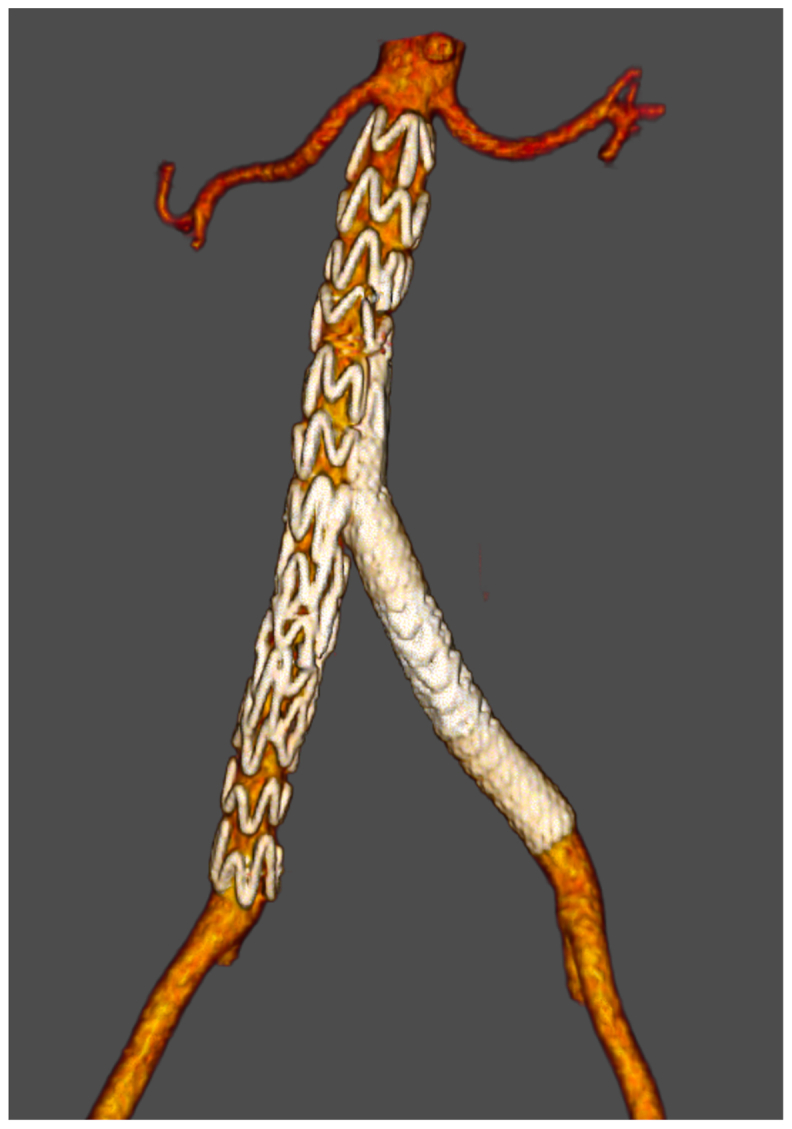
Thirty-day follow-up computed tomography (CT) angiography with three-dimensional reconstruction demonstrating complete aortic remodeling, preserved endograft patency, and restoration of normal aortoiliac anatomy.

The patient's recovery was uneventful, with complete resolution of abdominal pain and no hypertensive episodes. She was discharged 48 hours postoperatively in good general condition. Follow-up CT angiography at 30 days demonstrated complete remodeling of the dissected segment, with patency of both iliac branches and exclusion of the false lumens (Fig 3).

Written informed consent was obtained from the patient for the procedure and for the publication of this case and accompanying images.

## Discussion

This case represents an infrarenal dissection with progression to triple lumen, a finding that reflects extreme aortic wall fragility and has been associated with a poor prognosis.^1^ This sign, described as an indicator of dissection instability, represents the coexistence of multiple false channels and correlates with an increased risk of aneurysmal expansion and rupture. The presence of refractory pain in our patient reinforced the need for early endovascular intervention.

In the literature, the E-liac device has been shown to have consistent results in preserving hypogastric perfusion for the treatment of aortoiliac and isolated iliac aneurysms. Multicenter series have reported near 100% technical success, primary patency rates of 92% to 100%, and 1 -year survival of >95%.^3^, ^4^, ^5^ These results have established as a safe and effective strategy, reducing pelvic ischemic complications such as buttock claudication, erectile dysfunction, and colonic ischemia, which are more common when the hypogastric artery is sacrificed.

Beyond its original indication, off-label use of IBDs has been reported in complex anatomical scenarios. In 2019, Bozzani et al^2^ described the reconstruction of the aortic bifurcation using a Gore IBD, originally designed for hypogastric preservation in iliac aneurysms. This case demonstrated that an IBD can be adapted to replace an aortic body, allowing safe reconstructions in patients not treatable with conventional endografts.

Subsequently, in 2020, Orrico et al^6^ reported the aortic placement of a single E-liac in a patient with poliomyelitis-related aortoiliac deformities. In that scenario, conventional aortic endografts were unsuitable, and the E-liac device allowed successful repair thanks to its branched design and suitability for narrow bifurcations. This case was particularly significant, because it was the first report of this device being repositioned in the aorta. Our case further expands its applications by demonstrating successful off-label use in the setting of infrarenal aortic dissection, beyond the formal indication in aneurysmal or occlusive disease.

Our case adds to this line of innovation for the treatment of an infrarenal dissection with triple lumen and a narrow bifurcation. This strategy resolved a high-risk clinical situation, with complete reconstruction, preservation of bilateral flow, and favorable remodeling at early follow-up. These findings support the concept that, in selected anatomies and with meticulous planning, IBDs can serve as a valuable tool for complex aortic pathologies where conventional options are limited.

## Conclusions

Infrarenal dissection with triple lumen represents a high-risk condition that justifies invasive management when associated with refractory pain. The off-label use of the E-liac IBD in this case allowed successful endovascular reconstruction in hostile anatomy, with preservation of both iliac arteries and favorable early outcomes. This report, consistent with previous experiences, suggests that IBDs can broaden the spectrum of endovascular options in complex aortic disease.

## Data availability statement

All relevant data related to this case are included in the article. Additional details may be available from the corresponding author upon reasonable request.

## Declaration of generative AI and AI-assisted technologies in the writing process

Artificial intelligence tools (ChatGPT, OpenAI, September 2025 version) were used in the preparation of the English language and structure of the abstract and supporting text. The authors reviewed and edited the final version to ensure accuracy and appropriateness.

## Funding

None.

## Disclosures

None.
